# Vertical whole body vibration for treatment of knee osteoarthritis: a pilote monocentric prospective, randomized trial

**DOI:** 10.1007/s00402-025-05842-8

**Published:** 2025-04-11

**Authors:** Jan P. Hockmann, Kourosh Zarghooni, Gregor Stein, Kortessa Tersudi, Peter Knöll, Sebastian G. Walter

**Affiliations:** 1 Department of Orthopedic Surgery, Traumatology and Plastic Surgery, University Hospital Cologne, Cologne, Germany; 2 Department of Orthopedic and Trauma Surgery, Helios Clinic, Siegburg, Germany; 3 Department of Orthopedic Surgery and Traumatology, Klinikum Leverkusen, Leverkusen, Germany; 4 Joseph-Stelzmann-Str. 24, 50931 Cologne, Germany

**Keywords:** Osteoarthritis, Knee, Whole body vibration, Physiotherapy, Pain, Stiffness, SF-12, WOMAC

## Abstract

**Introduction:**

Osteoarthritis of the knee (KOA) is a leading cause of disability in the aging population. The treatment of choice in most stages is a conservative multimodal approach. Previous studies were able to prove the efficiency of physical therapy for improvement. Therefore physical therapy, besides pain medication, is one of the most common used forms of therapy for KOA. This study aims to evaluate the possible efficiency of whole-body vibration (WBV) compared to physical therapy. This might benefit patients to whom physical therapy is not accessible.

**Materials and methods:**

Patients with primary Gonarthrosis grade II or III were recruited. Included patients were randomly allocated to two groups. One group was treated by physical therapy and the other one with WBV. Treatment duration was six weeks. An Intention-to-Treat analysis was performed. Effectiveness was evaluated by Western Ontario and McMaster Universities Osteoarthritis Index (WOMAC), Outcome Measures in Rheumatology Committee (OMERACT) and Short-Form-Health-Survey 12 (SF-12) at seven, twelve and 26 weeks.

**Results:**

Of 51 patients recruited, 39 patients were finally included. Overall, both treatments were able to show improvements. The SF-12 Score was improved in both groups without significant difference (*p* = 0.487). The conventional group showed insignificant vaster pain reduction (*p* = 0.926). Whereas WBV resulted in insignificant improved function (*p* = 0.144), reduced stiffness (*p* = 0.931) and improved total score (*p* = 0.295). Response to therapy reduced over time in both groups. Although more patients of the WBV group reported improvement of their general health status, average improvement was better at the conventional group.

**Conclusions:**

This study was able to show that, for the conservative treatment of knee osteoarthritis grade II and III, WBV is a non-inferior therapy compared to conventional physiotherapy. Both were able to improve the status of the patients and may be used based on the accessibility and preferences of affected patients.

## Introduction

One of the most common causes for pain and impaired function at the musculoskeletal apparat is osteoarthritis of the knee (KOA) [1]. The global, aging population is contributing to over 240 million people affected by osteoarthritis [2]. In 2007, approximately 14 million people were affected by KOA in the United States of America [3]. Age and Body-Mass-Index (BMI) have been identified as risk factors [4]. Treatment includes oral pain medication with non-steroidal anti rheumatic drugs (NSAR), physical therapy, infiltration therapies and in advanced, severely symptomatic cases arthroplasty is the treatment of choice [5, 6]. The main goals of treatment are pain reduction, restoration of knee functionality and improvement of quality of life. Basic treatment aims at reducing risk factors such as reducing body weight and maintaining knee-joint function by strengthening muscular joint guidance [7]. As early as 1950 whole body vibration training (WBV) was described for medical treatment purposes [8]. Nowadays it is an established technique in settings where muscular strength as well as proprioception are trained and needed. Studies have shown its effectiveness as a postoperative recovery possibility [9–11]. In a mouse-model of knee osteoarthritis cartilage and subchondral trabecular bone were preserved after exposure the WBV [12]. A recent meta-analysis showed that WBV together with strengthening exercises had additional positive effects compared to exercises alone [13]. A study with 30 participants showed that athletes with patellofemoral pain could improve pain and performance equal to physical therapy [14]. Most studies to date compare WBV as an addition to physical therapy [15]. Given the challenges e.g. of recent quarantines during COVID 19, home exercises are evaluated even more as they get more popular [16].

This study evaluates the therapy as a standalone therapy in a randomized controlled study compared to conventional physiotherapy.

## Methods

### General

**Fig. 1:**
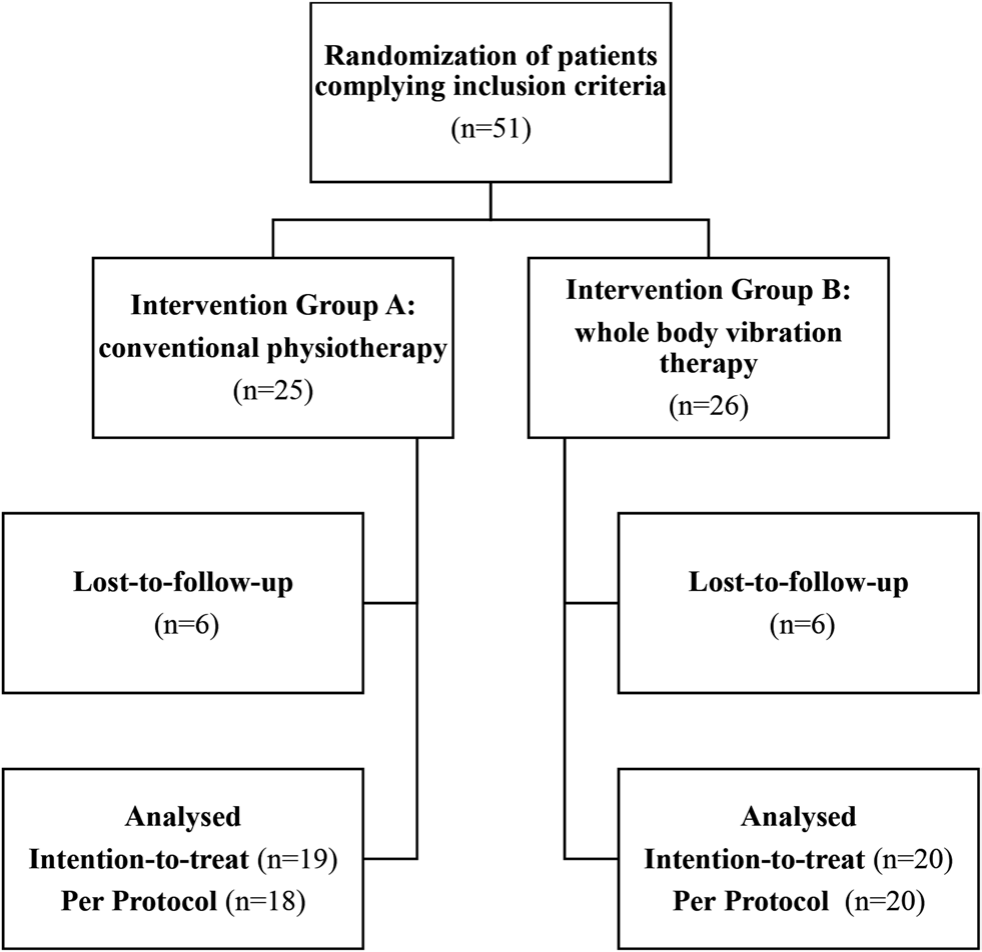
Consort Diagram of patient randomization

There were 51 voluntary patients recruited by advertisement in the newspaper, internet and posters. Patients were allocated to either physiotherapy or WBV by a randomization sequence, which is illustrated in Fig. 1. Treatment included training three times a week for six weeks. The sessions were supervised by physiotherapists. Each patient was observed for six and a half months. There were five visits with each patient of which three were follow-ups at seven, twelve and 26 weeks. The WOMAC questionnaire, which has previously been validated for patients affected by osteoarthritis, was used for evaluation [17, 18]. We standardized the WOMAC data to a scale of 0 to 100 with 0 referring to perfect articulation and no symptoms. Outcome Measures in Rheumatology Committee (OMERACT) were used to evaluate the responsiveness to the treatment. They are based on the WOMAC and have been used in several studies [19, 20]. Quality of life was evaluated by the German version of the Short-Form-Health-Survey 12 (SF-12) [21, 22]. For comparison of the psychological and physiological scores of the SF-12 to the general public the score of each patient was compared to the average score of the same age group [23].

Global health status of the patients was assessed by five possible answers ranging from very bad (0 points) to very good (5 points).

First screening of the patients was performed to ensure inclusion criteria were met and exclusion criteria were absent. Therefore, the patient reported outcomes (PROM) were also recorded for the baseline examination by TS.

Inclusion criteria were diagnosis of a with uni- or bilateral primary Gonarthrosis grade II or III according to Kellgren and Lawrence [24], age between 30 and 80 years, Body-Mass-index below 40 kg/m^2^.

Exclusion criteria were a WOMAC pain scale over 70 mm, active physiotherapy within the last 6 months or previous surgery of the affected knee or secondary osteoarthritis.

Then the treatment started for consecutive six weeks according to the two groups. Conventional intervention group: The conventional intervention group performed approximately 30 min of physiotherapy for knee joint guiding muscle groups with 40–50% of maximal power, 15–20 repetitions with three to six sets. Treatment further included stretching, manual therapy, improvement of coordination, training of daily life situations and treatment of adjacent joints. Whole-body-vibration intervention group: The WBV group was treated with the Galileo^®^ (Novotec Medical GmbH, Pforzheim, Germany) whole body vibration device based on a predefined schedule (Picture 1). Each training involved six sets each lasting three minutes. There were three phases. Phase one included initiation of muscle tension, phase two improvement of muscular power and phase three body coordination.

At week seven, twelve and 26 follow-up of the patients was performed and the PROMS were recorded at each time by KT.

**Picture 1:**
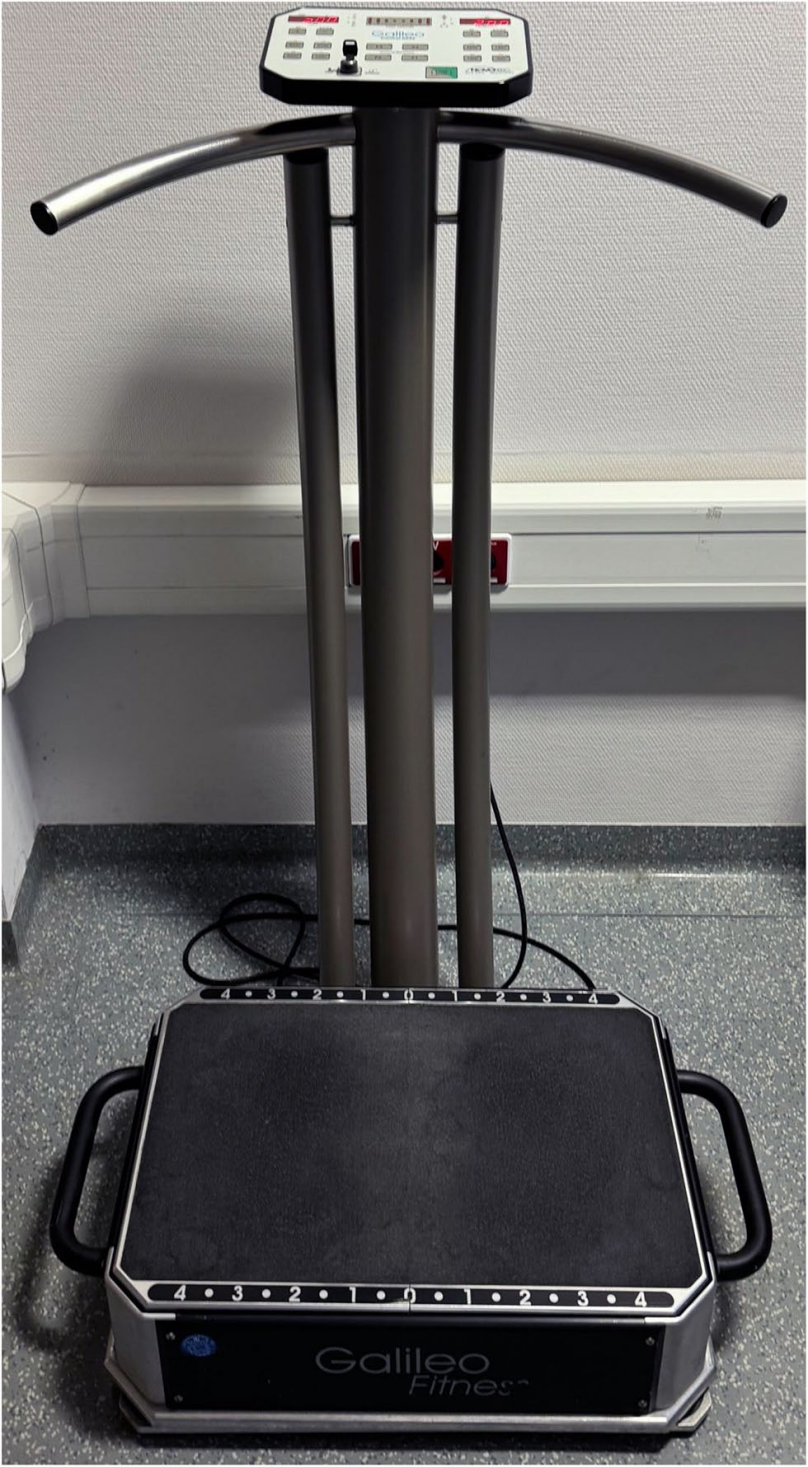
Galileo^®^ (Novotec Medical GmbH, Pforzheim, Germany) whole body vibration device as used in the study

### Statistical analysis

The sample size was calculated based on a standard deviation/significant difference of the therapeutic effects for both groups measured by WOMAC of σ = 19 derived from literature [25]. With the requirement of an 80% power, the sample size needed was 31 patients and a clinically significant difference starting from 20 on the scale of the WOMAC was determined.

The WOMAC was evaluated with SAS 9.2. An analysis of variance (ANOVA) and analysis of covariance ANCOVA was used. For the OMERACT-criteria Fisher’s exact analysis was applied. The SF-12 was evaluated by IBM SPSS Statistics Version 20.0 for Microsoft Windows (IBM Corp, Armonk, NY). Wilcoxon signed-rank test, Mann-Whitney-Test, Kruskal-Wallis-Test as well as ANCOVA were used. We report average ± standard deviation and median with range (minimum–maximum). A value of *p* < 0.05 was considered to be statistically significant.

## Results

### Cohort characteristics

39 patients were included for Intention-to-Treat analysis. Demographic data are summarized in Table 1. The conventional group had a median age of 58.6 (31.9–72.4) years, the WBV group of 63.95 (35.1–73.9) years.

**Table 1:** Age and gender distribution according to intention-to-treat

Age and Gender distribution according to intention-to-treat	value
Age, median (minimum-maximum)	
Total Conventional group WBV group	60.60 (31.90–73.90) 58.60 (31.90–21.40) 63.95 (35.10–73.90)
Sex total, n (%)	
Male Female	16 (41.02) 23 (58.97)
Sex conventional group n (%)	
Male Female	7 (36.84) 12 (63.16)
Sex WBV group, n (%)	
Male Female	9 (45.00) 11 (55.00)
WBV = whole body vibration	

### WOMAC

The average total improvement of the WOMAC-Scores is depicted in Table 2.

**Table 2:** Changes of the WOMAC-Scores

Group	*N*	Score	*N*	average	SD	Min.	Max.
Physical therapy	19	pain function stiffness global	14 13 14 13	-8.78 -0.12 -7.25 -2.69	17.1 21.19 22.45 18.61	-41.86 -46.31 -48.85 -44.02	21.46 29.78 24.5 22.04
Whole body vibration	20	pain function stiffness global	15 15 13 12	-8.2 -11.69 -7.9 -10.28	16.04 17.01 15.65 16.63	-46.14 -59.44 -41.85 -54.09	10.8 13.0 16.0 9.59

N = number, SD = standard deviation, min.=minimum, max.=maximum

For the conventional group, greatest reduction of pain, function, stiffness and the global WOMAC was observed after twelve weeks. Pain reduced by -9.16 ± 4.44, function by -4.64 ± 5.32 and stiffness by -11.05 ± 5.05. Best total WOMAC reduction achieved in the conventional group was − 6.51 ± 4.68.

Whereas best improvement in the WBV group was achieved after six weeks for pain by -8.12 ± 4.24. Function and stiffness improved best after twelve weeks by -12.03 ± 4.69 and by -13.05 ± 5.15. Best total WOMAC improvement was observed after twelve weeks by -11.48 ± 4.48.

Pain showed a bigger difference in the conventional group. Function, stiffness and total score had a bigger difference in the WBV group. There was no statistical significance between the groups. Not for WOMAC stiffness (*p* = 0.784), function (*p* = 0.305) nor total (*p* = 0.450).

### Global status

At the start of the study the average global status was 3.3 ± 0.5 for the conventional group.

Best improvement was reached after six weeks with 3.7 ± 0.5. Improvement was reported by 21% of the patients in this group.

At baseline the global status in the WBV group was 2.8 ± 0.7. The best improvement was also after six weeks up 3.6 ± 0.7. In total 34% of the patients in the WBV group reported improvement in global status.

### OMERACT-OARSI-Response-criteria

Results of the OMERACT-OARSI-Response-criteria are summarized in Graphic 1. After six weeks, nine strict responders were reported for the WBV group in contrast to seven in the conventional group. In both groups these numbers reduced over time. There was no significant difference at any time. There were four patients with consistent response at all follow-ups in both groups.

**Graphic 1:**
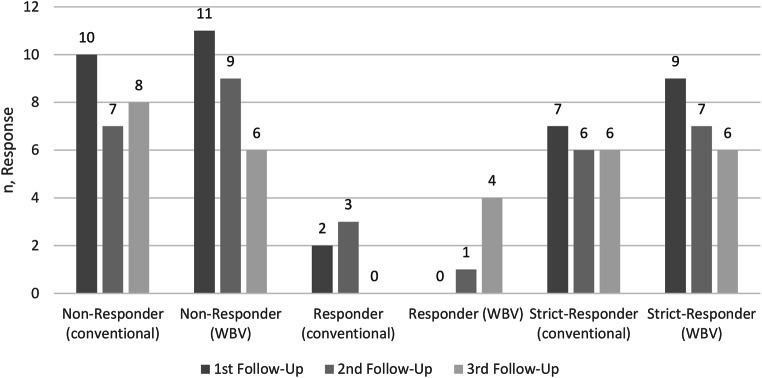
OMERACT-OARSI-Response-criteria results

### Quality of life based on SF-12

Graphic 2 shows the average values of physiological and psychological scales at the beginning and throughout the follow-ups. The biggest difference was achieved after twelve weeks with physical scale gaining 4.62 and psychological scale gaining 4.16. The scores improved at all times of follow-up. For the average physical scores, the conventional group showed a score of 39.85 ± 7.64 at base line and the WBV group a score of 36.29 ± 7.37. Best improvement was found in the WBV group, after twelve weeks by 6.91 points to 43.83 ± 12.24. The conventional group showed an increase at that time by 3.98 to 42.38 ± 5.7. The changes proved not to be statistically significant (*p* = 0.052).

**Graphic 2:**
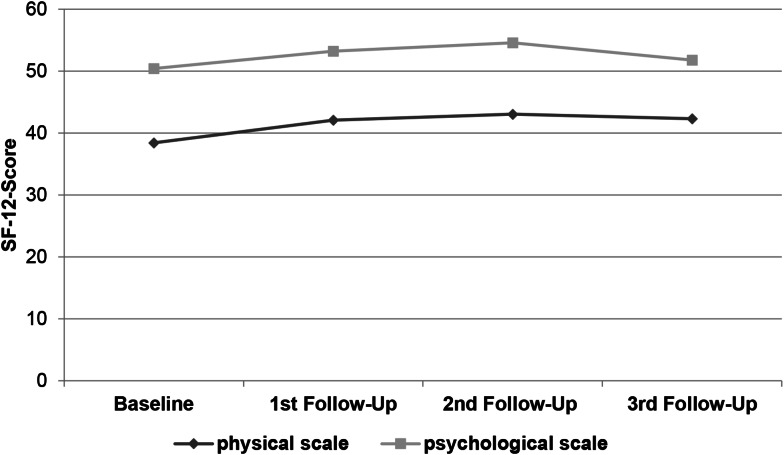
SF-12 scores during follow-up

The psychological average scale showed best improvement after twelve weeks. The conventional group improved by 4.41 points and the WBV group improved by 3.82 points. Differences showed not to be statistically significant.

Graphic 3 displays the average values for the average score of the physical and psychological score of both study groups compared to the general public scores. All scores were lower in the study population. The difference between the WBV group 36.9 ± 7.4 and general population 45.7 ± 4.0 was 8.8. For the conventional group 39.9 ± 7.6 the difference to the general public 46.3 ± 3.5 was 6.4 points. No differences were statistically significant.

**Graphic 3:**
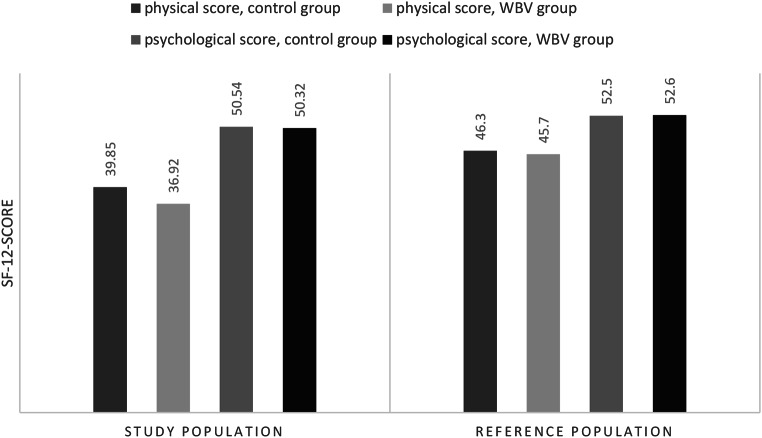
SF-12 scores for study population and general public

## Discussion

This prospective, randomized controlled study analyzed patient related outcome measure after conventional physiotherapy versus whole body physiotherapy in patients with knee osteoarthritis. To the best of our knowledge this the first study investigating this research question.

The study found that (especially after twelve weeks) improvements in all patients were found. Some scores showed greater improvements for patient reported outcomes within the WBV group, but differences were never significant. The average age difference between both groups, that was statistically significant, may have an influence on the results as loss of function, muscle strength and other influencing factors increase with age. Analysis of the WOMAC score found pain reduction, reduction of joint stiffness and improvement of function for both groups. A study evaluating age differences in the effect of WBV did not show significant differences [14].

The results of the presented study suggest pain reduction in the WBV group after six weeks. This coincides with findings of another study observing pain reduction after one WBV session. They concluded a relation between improved function and pain. Our data supports these findings as function improved [26].

Studies were able to prove that WBV as an addition and alone is equivalent and for some outcomes improves conventional physiotherapy alone in patients with patellofemoral pain. Improvements of flexibility by adding WBV to conventional physiotherapy for patients with patellofemoral pain was observed [15]. Shadloo et al. could prove that WBV had the same positive effects on patellofemoral pain as conventional physiotherapy [27]. They also had similar sizes of study population as this study. Similar group sizes were also achieved in a study evaluating the effects of WBV in female patients with knee osteoarthritis [25]. The results of those studies are in accordance with the findings presented in this study.

Although treatment was performed for six weeks only improvements were observed also after 26 weeks. A meta-analysis showed significant improvements after treatment for eight and twelve weeks for function but no significant improvements for stiffness and pain measured by WOMAC [28]. If longer treatment would result in different outcomes remains unclear.

The subjective well-being of the patients measured by SF-12 as well as global status did not decrease during the treatment which might lead to perseverance of patients treating knee osteoarthritis conservatively.

OMERACT-OARSI-Response-criteria showed especially results of strict or non-responders in both groups. The small proportion of responders may be due to gender or Kellgren and Lawrence stage as in both groups there were two characteristics involved. Evaluation of those subgroups were not included but the data may suggest influence of these factors. One might assume more severe form of osteoarthritis may lead to better or worse resonance to the treatment. The WBV group had more responders and strict responders at the final follow-up. Long-term effects are important in treatment of symptoms of knee osteoarthritis as this may encourage patients to maintain conservative treatment as long as possible. Although radiographic progress of the disease was not examined and statements regarding the progress of the disease on a long term basis cannot be performed.

### Clinical implications

This study shows that the WBV effectiveness is not inferior to physiotherapy with an instructor. However, WBV yields several advantages. For an individual physiotherapy cycle as described a physiotherapist would be needed for in total nine hours. For the WBV training a physiotherapist would only be needed once only to instruct the patient and subsequent therapy could be performed by the patient himself. A study evaluating the combination of at-home training with a WBV device and physiotherapy in children was able to show good compliance and improvements [29]. Park et al. were able to show that home treatment with WBV devices in knee osteoarthritis proofed to be effective [30].

### Limitations

The presented study is limited by its single center design, short follow-up, short treatment period and small study population. Insignificant differences between groups may have become relevant within a larger study population. Long-term studies should evaluate time-to-decision for arthroplasty after conservative treatment with physiotherapy and WBV. Another limitation is that the patients were not blinded and recruited especially for this study. Therefore, results might be subjective.

## Conclusion

This study demonstrated that WBV is not inferior to conventional physiotherapy for the treatment of knee osteoarthritis with respect to patient rated outcome measures. Therefore, WBV might be considered as optional pillar for multi-modal conservative treatment in knee osteoarthritis.
